# Mass email risk communication: Lessons learned from COVID-19-triggered campus-wide evictions in Canada and the United States

**DOI:** 10.1371/journal.pone.0266242

**Published:** 2022-04-05

**Authors:** Haorui Wu

**Affiliations:** School of Social Work, Dalhousie University, Halifax, Nova Scotia, Canada; Universitat Luzern, SWITZERLAND

## Abstract

From an out-of-province/state and international post-secondary student perspective, this article (a) explores mass email risk communication facilitation during the COVID-19-triggered campus-wide evictions in Canada and the United States; and (b) develops relative recommendations to improve mass email risk communication strategies for future emergency response. Investigating mass email risk communication-related impacts on students in a tertiary educational context has revealed a significant deficit in emergency response research, practice, and policymaking. Mandatory temporary university and college closures during the COVID-19 first wave provided an opportunity to address this research and practice deficit, as most Canadian and American universities/colleges administered their eviction communication via daily mass email chains. Through a phenomenological lens, this study interviewed twenty out-of-province/state and international students, ten from each country respectively, to examine student eviction experiences associated with intensive mass email risk communication. This research identified four factors linked to mass email risk communication: email chain characteristics, student interpretation, interdepartmental cooperation, and frontline voices. Synthesizing these findings, four evidence-based recommendations were developed: to efficiently convey risk information to students, to understand student perceptions and to inform their behaviors, to enhance interdepartmental cooperation, and to enable mutual dialogue in decision making. These recommendations could assist post-secondary institutions, and other organizations, in strengthening their mass email risk communication strategies and advancing organizational emergency response plans for future extreme events.

## Introduction

The global COVID-19 public health emergency response reinforces a well-known risk communication dichotomy, where communication strategies serve to either inform the general public [1, 2] or create more chaos [3, 4]. Widespread coronavirus and variant transmission have directed attention towards institutions/organizations with highly vulnerable populations, such as long-term care facilities [5], homeless shelters [6], and schools [7]. In a post-secondary educational context, current COVID-19-specific research has predominantly focused on various pandemic impacts (e.g., social, health and well-being, economic, and operation) at both individual and organizational levels [8, 9]. Although mass email serves as one of the principal campus-wide communication approaches, literature that explores campus-wide mass email risk communication impacts, especially relating to emergency response and eviction, is limited.

During the pandemic first wave (commencing March 2020), temporary university/college closures initiated immediate on-campus student evictions, engendering tremendous challenges for out-of-province/state and international students in both Canada and the United States (U.S.) [10]. In April 2020, a quick response disaster research partnership was developed to explore undergraduate and graduate out-of-province/state and international student eviction experiences in both countries [11]. Using in-depth interviews, this study collected perishable data regarding student eviction experiences, intending to identify various challenges facing students during the campus-wide eviction process, including any solutions they deciphered, to improve university emergency response planning for future extreme events.

Most universities/colleges utilized multimedia strategies (e.g., posters, website announcements, and social media) to widely distribute eviction information and COVID-19 specific updates, however intensive mass email is still one of the primary university/college risk communication administrative approaches [12]. Although risk communication was not the original intent of this study, various challenges associated with mass email risk communication emerged as a prominent theme throughout the data analysis process, inspiring this article. Specifically, this article explores how structure, composition, and other related factors inextricably linked to mass email risk communication, influenced student eviction experiences. Based on these findings, mass email risk communication recommendations were provided from a student perspective to improve future emergency responses, particularly applicable in a tertiary educational setting, while more generally relevant to organizations with highly vulnerable populations.

## Mass email risk communication, COVID-19, and tertiary education

Mass email communication, an Internet-based information communication strategy that delivers a single message to a large group of stakeholders, has radically reshaped the traditional communication landscape across multiple sectors, including health care and social services [13, 14], retail [15], public administration [16], political campaigns [17, 18], and academia [19]. In the disaster and emergency management field, mass email communication fundamentally supports risk communication [20], featuring salient, real-time information exchange between professionals and the general public, aiming to reduce physical, social, economic, and other losses, and enhance inhabitant physical health, mental wellness, and overall well-being [21]. This literature review section will examine existing mass email risk communication academic publications in a COVID-19 and tertiary educational context.

### Mass email risk communication and COVID-19

For decades, researchers, practitioners, policy decision-makers, and other stakeholders have thoroughly explored risk communication in response to three major hazard types: natural hazards [22], technical accidents [23], and terrorist attacks or other intentional violence [24]. Corresponding research investigates various risk communication-related factors, including risk communication approaches (e.g., mass media and cutting-edge social media) [25] and communication among different user groups (e.g., adolescents and ethnic minorities) [26]. Most current studies contribute to in-depth analyses regarding the interplay between risk communication and risk-driven behaviors associated with demographic factors, socioeconomic status, and other distinguishing intersectional characteristics [27]. These studies have made such contributions by evaluating different risk communication approaches, especially pertaining to efficacy, through identifying strengths and weaknesses in conveying vital and correct information to target populations. Studies concerning risk communication in eviction and evacuation processes, particularly in tertiary education, remain inadequate.

The global COVID-19 public health emergency has led to a comprehensive examination of emergency response systems at the community, provincial/state, and national levels [28]. Nascent research regarding COVID-19-based risk communication has focused on health consequences, particularly in medical and public health domains [29, 30]. Risk communication approach evaluations have also aligned with healthcare missions, studying efficient and precise public health information provision strategies to inform and educate the general public, preventing pandemic spread [31, 32]. Although COVID-19 significantly impacts educational organizations, current research regarding organizational risk communication has followed a similar health-focused trajectory, rather than addressing effectiveness and end-user impacts [33].

Throughout the COVID-19 pandemic, societal risk communication-related topics such as top-down administration and cooperation [34], misinformation [35], public trust [36], stigma, and mental health [25], continue to emerge via academic studies, particularly associated with social media [37] and mass email communication approaches [38]. Indeed, Yong and colleagues [39] discovered that secure text messaging, rather than email, played an essential role in effective emergency risk communication in a Singapore hospital setting. Klich [40] argues that a lack of electronic communication regulations (including email) increased misinformation spread within Poland’s COVID-19-specific public health strategy. As email is one of the principal communication approaches in post-secondary educational organizations [41], integrating both public health and societal dimensions into risk communication in a tertiary educational context has revealed a significant gap in both research and practice. Namely, a lack of research focuses on the effectiveness of mass email in facilitating campus-wide risk communication, especially in response to temporary campus shutdowns during the first wave of COVID-19.

### Mass email risk communication in post-secondary education

In post-secondary educational organizations, mass email communication critically connects and engages students, faculty, and staff in diverse academic and related activities [14]. For example, faculty members increasingly indicate that mass email communication is an effective teaching tool, and students illustrate positive ways to incorporate email into their learning process [42]. Zaid and colleagues [43] provide I.T. system strategies to improve automatic malicious spam detection to support effective communication among campus-wide stakeholders. In comparison to current mass email communication industry approaches, Dawkins [41] argues that academia-related mass email evaluation and optimization strategies are under-researched, especially relating to identifying existing barriers to mass email communication effectiveness. In particular, mass email commination has not been comprehensively evaluated in a campus-wide disaster risk communication scenario.

Previous research concentrates on utilizing mass email as an information outlet in disaster settings [20] or employing mass email as a data collection instrument to address extreme event consequences [44, 45]. Studies have not comprehensively explored the role of email as an information facilitation tool during the emergency response stage or the associated impacts on extreme event affected populations. As a preferred method of professional communication, higher educational institutions have developed detailed guidelines to improve email etiquette, efficacy, and effectiveness among campus-wide users [12, 46]. Enabling mutual dialogue is recognized as one of the most effective practices to promote communication and enhance mutual understanding, an approach that could be seamlessly applied in a disaster context [47]. However, university/college administrative risk communication emails were delivered through a top-down approach, typically without engaging frontline voices (e.g., students, staff, and faculty).

Furthermore, extreme events increase the degree of complexity in composing effective email content for risk communication. Notably, as early as the 1960s, Anderson [48] identifies the unpredictable nature of disaster and inadequate information access as significant challenges to maintaining public willingness to follow emergency evacuation instructions. Similarly, many students may not quickly identify salient information, with some perhaps swayed by conflicting information [49]. Conflicting information might lead students to overestimate potential negative eviction impacts (e.g., depression and anxiety) or cause situational misjudgment [50]. These challenges are also reflected in campus-wide risk communication via emails.

According to Byron [51], email recipients frequently apply emotional negativity towards administrative emails, a response incongruent with sender-intent. In combination with extreme event stress, this effect might significantly affect student, faculty, staff, and other on-campus resident mental health and overall well-being [52]. Although email is one of the major pandemic risk communication approaches [41], current research rarely examines email communication-related health and well-being impacts on various on-campus stakeholders. Furthermore, as emergency responses require multi-sector collaboration, email communication facilitates the emergency response process through multiple authorities within the university system [53]. Evaluating email risk communication during COVID-19 could improve multi-stakeholder collaboration towards on-campus emergency response and advance emergency response planning in higher education. Merging these factors highlights research deficits relating to email risk communication evaluation during the COVID-19 driven emergency response.

The COVID-19-triggered campus-wide eviction generated a valuable opportunity to address the aforementioned research deficits. As the email chain, at best, connects students with university authorities during the eviction process, identifying evidence-based recommendations will promote risk communication operational efficiency and advance university-specific emergency responses. Hence, from an out-of-province/state and international student perspective, this study is guided by the following four questions: (1) What were the mass email chain characteristics during the pre-, peri-, and post-eviction stages? (2) How did students interpret these email chains? (3) What were the other on-campus community authority and stakeholder contributions towards the email-facilitated eviction? (4) What improvements can be made to student-specific email risk communication strategies that would better serve on-campus and extended communities during the emergency response stage?

## Research design

This study utilizes a phenomenological lens to qualitatively examine out-of-province/state and international university/college student eviction experiences during the COVID-19 first wave in the U.S. and Canada [10, 11]. Ten students from both countries, respectively, were recruited through both convenience and snowball sampling approaches. Perishable data were collected through in-depth interviews. The research ethics application of study was approved by (1) the Social Sciences and Humanities Research Ethics Board at Dalhousie University, Canada (Certificate Number: 2020–5371) and the informed verbal consent was obtained from each Canadian participant; and (2) the Institutional Review Board at the University of Puerto Rico Mayaguez, the U.S. (Protocol Number: 2020040014), which waived the requirement to obtain informed consent for all the U.S. participants.

### Participant recruitment

Aiming to collect time-sensitive data, virtual invitations were distributed through various student contact lists, social media, and online advertising platforms (e.g., Facebook) to reach a broad segment of potential participants in both Canada and the U.S. Potential participants were asked to self-confirm their eligibilities according to the following two criteria: students who (1) identify as out-of-province/state students or international students according to each country’s immigration definitions, and (2) were evicted from their on-campus residency due to COVID-19-triggered campus closures. The first ten students in each country (20 in total) who self-determined their eligibilities were invited for in-depth individual interviews.

Generally, out-of-province/state or international students have fewer local social ties than their in-province/state peers [54–56]. As such, when eviction orders were issued, the team hypothesized that out-of-province/state or international students might have had difficulty quickly identifying local accommodations, or, with fewer social ties than their in-province/state peers, may have struggled to identify and navigate local resources to arrange a swift return to their original homes. Moving back home became extremely difficult, even risky, and unaffordable for some international students [57, 58]. Hence, this study focused on out-of-province/state or international students as they were confronted with more challenges than their in-province/state peers. Addressing these challenges would enable university/college authorities to improve current emergency planning strategies to better prepare for future extreme events.

Participant demographics in both countries reflect diversity across various domains. Representing different facets of academics, participants from both countries were enrolled in both undergraduate and graduate programs at both public and private research-intensive universities, of which 50% of Canadian participants and 90% of American participants were undergraduate students. Female students represented 55% of the total participants from each country (six in Canada and five in the U.S.). Among the 20 participants, 12 identified as ethnic minorities (six in each country, respectively). Five of ten Canadian institution participants self-identified as international students, whereas only one international student was represented among the American institutions.

#### In-depth interviews

All 20 in-depth interviews were completed within one month (May–June 2020). Interviews were audio-recorded, each spanning 45 minutes to one hour. The country-based interviews were conducted through virtual conference software, using Microsoft Teams (Canada) or Zoom (the U.S.), respectively. Participant eligibility was verified before engaging in any research activities, and a verbal consent was obtained at the beginning of each interview. Participant recruitment letters and interview protocols (including interview questions) can be accessed from the DesignSafe-CI data repository (https://doi.org/10.17603/ds2-wxsx-qs30) [59].

Students answered open-ended interview questions, divided into two sections. The first section focused on participants’ basic information, such as educational program (e.g., major and undergraduate or graduate) and motivations behind their program of choice (e.g., career aspirations and academic goals). This background information is foundational to contextualizing data analysis in the participants’ educational context. The second section pertained to participant eviction experiences. Interview questions were developed to encourage participants to recall eviction memories from the following three stages: pre-eviction (when COVID-19-specific public health restrictions were in effect), peri-eviction (when universities/colleges issued eviction orders), and post-eviction (when participants moved out of their on-campus housing). Detailed interview questions were designed to identify eviction-related challenges and participant-developed solutions, including any external supports they received, and also to capture the eviction process-driven impacts on mental health, academic planning, and social lives. Interviewers carefully documented observation notes and requested clarifications during the interview process to comprehensively and accurately capture participant eviction experiences.

### Data analysis

According to qualitative research standards, a sample size of 20 participants supports thematic analysis in a homogenous group of university/college students [60]. All 20 interviews were transcribed and imported into NVivo 12 software (QSR International, Melbourne, Australia) for thematical analysis (coding and theming). Utilizing an interpretive paradigm [61], a content analysis approach identified broad themes associated with participant eviction experiences [62]. The author conducted two rounds of data analysis through deductive (top-down) and inductive (bottom-up) strategies.

Following the interview flow, the first-round of data analysis deductively identified the following four sub-categories: challenges (e.g., obtaining information and off-campus housing searching), solutions (e.g., seek clarification and collect belongings), support (e.g., family, friends, and supervisors), and various impacts (e.g., mental health, adverse coping behaviors). Although mass email risk communication evaluation was not the original intent, it clearly emerged as a theme during this initial round of data analysis and is strongly associated with all codes and sub-categories. This connecting thread woven throughout the data motivated the author to conduct a second round of data analysis to comparatively examine the interplay between mass email risk communication and participant eviction experiences.

**Fig 1:**
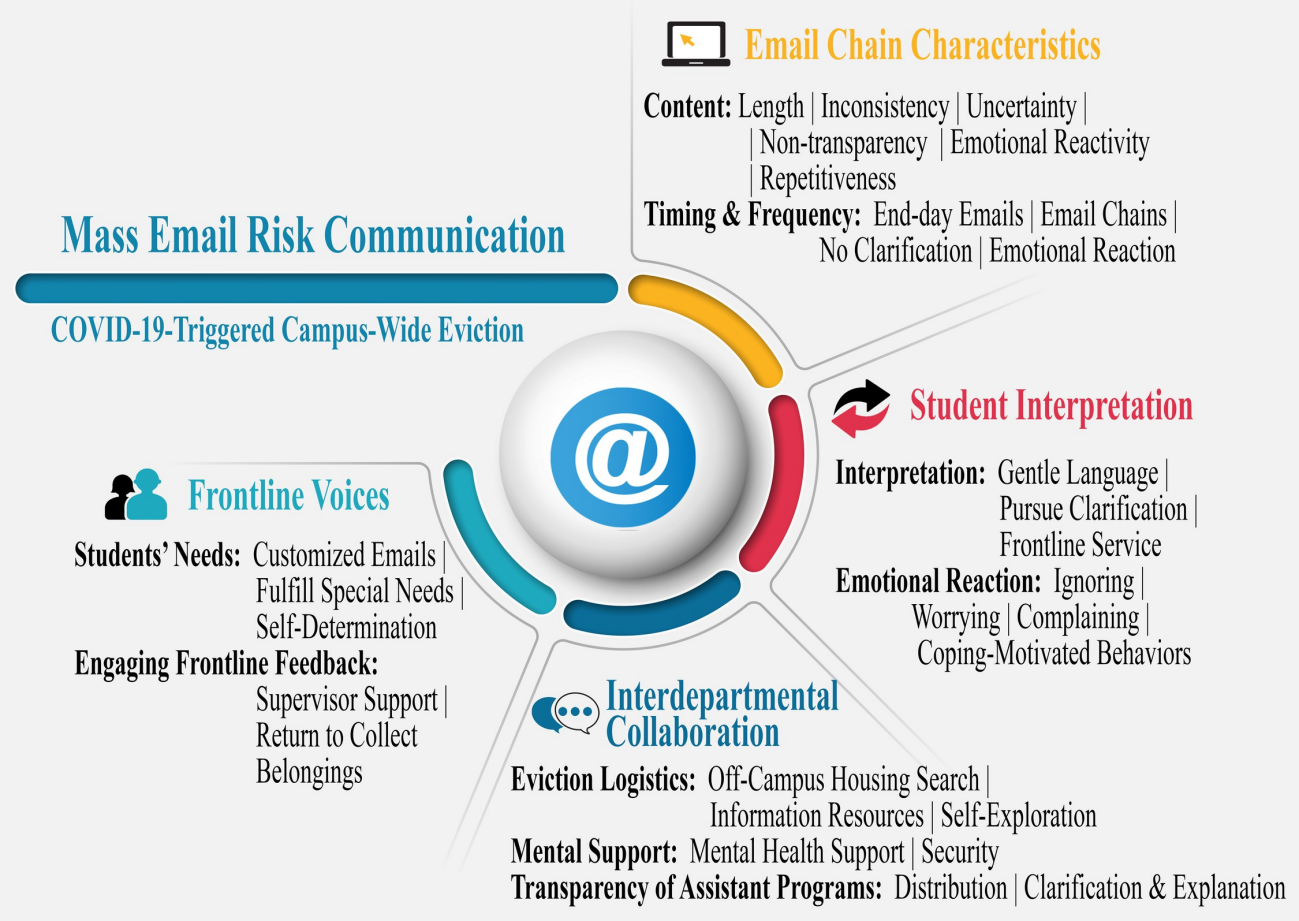
Codes and themes for mass email communication. The four sections (Email Chain Characteristics, Student Interpretation, Interdepartmental Collaboration, and Frontline Voices) demonstrate the four themes that emerged in the data analysis regarding out-of-province/state and international student eviction experiences. Under each theme, bi/tri-level sub-categories were developed to provide detailed supporting information. Each subtheme was followed by different codes used to identify related information from interview transcripts.

Based on the outcome of the initial top-down data analysis, the second-round analysis adopted a bottom-up inductive approach to understand mass email risk communication roles during the entire eviction process [63]. Although the email eviction order signaled the beginning of the eviction process, administrative risk communication began before the eviction order, coinciding with the initial COVID-19 surge in North America. Furthermore, after most students had moved out of their dormitories, ongoing email communication kept them informed on campus news. Hence, the chronological dimensions of pre-, peri-, and post- evictions were captured during this round of analysis to examine the dynamic connections made throughout the entire risk communication process. As shown in Fig 1, four themes (Email Chain Characteristics, Student Interpretation, Interdepartmental Collaboration, and Frontline Voices) were developed. Under each theme, sub-categories were supported by different codes.

## Findings

Based on the high frequency of emails administrated during the pre-, peri-, and post-eviction stages, this section will present finding through the four elements shown in Fig 1. These elements aim to promote the eviction process mass email risk communication operational efficiency. Each element was expanded through different sub-categories, supported by participant eviction experiences.

### Element 1: Characteristics of email chain

The fast-evolving COVID-19 pandemic significantly increased the unpredictability of risk communication [35]. This section will evaluate mass email effectiveness from two aspects: content and timing/frequency.

#### Content

Brevity, clarity, consistency, and transparency are campus-wide email communication fundamental principles and are essential components of effective risk communication [12]. All participants indicated emails received during the eviction process were significantly longer than those sent during non-emergency times. Moreover, email language was vague and uncertain. These email characteristics posed challenges for students, staff, and faculty, creating barriers for email recipients to quickly and easily capture core messaging and avoid confusion at the initial critical communication stage. As a U.S. participant described:

*I think it’s like 33 pages that they [university] sent us about recommendations, like things [they] were planning to do, which was good. But at the same time, they [university] still didn’t know what was going to happen. We were all very confused at the beginning*.

*Every student already suspected it [eviction] because, in the same morning, Harvard said they were going to kick students out. Everybody already knew what’s going to happen that same afternoon, they sent out a pretty long email just saying, “Hey, you guys need to be out within a week.”*

During the COVID-19 first wave in Canada and the U.S., rapid daily rising cases, along with widespread misinformation, triggered student anxiety and raised concerns relating to screening correct information. University administration official risk communication channels were expected to provide reliable information; however, their unclear and inconsistent outgoing emails were insufficient to adequately address student needs.

A U.S. participant specified, “*It seemed like every email was repeating itself*. *So*, *I was just feeling very anxious*, *waiting for the next instructions*, *the next rules*. *There were no clear steps of what we had to do*.”

A Canadian participant indicated: “It’s *called an email chain*, *right*? *It’s really long and difficult to follow*. *We just need a very brief*, *certain email telling us what to do*. *One told us to do A*, *but the next one told us to do B*. *I could not figure out what the university really wanted us to do*.”

Upon campus closures, all in-person instruction continued virtually. However, field education and internship postponements interrupted scheduled graduations for some final-year students. As an illustration, when their university issued the eviction order, one Canadian participant involved in field education, who had only one week left to fulfill the degree requirements, described:

*One email said that we could apply for an extension with ‘uncontrollable reasons.’ I was hoping to finish the last week of my placement and then return home to find a job. I applied for an extension, but I was told that my placement was not one of the ‘uncontrollable reasons.’ It seemed that the university did not make all the information transparent*.

Working as a resident assistant in a residence hall, one Canadian participant was concerned these problematic emails caused negative impacts on frontline staff.

*Most staff were very worried because, like us, they really didn’t know what the university expected. They had to answer students’ questions, but they were very confused. I saw my superiors were so stressed. I presented to be normal, like nothing happened. If other students saw I was worried, they would feel even worse*.

### Timing and frequency

Generally, university communication protocol requires that every email undergo an interdepartmental review, which causes email delays during the eviction process. Consequently, most participants indicated that daily eviction-related updates were emailed to students at the end of each day when most on-campus employees were unavailable to provide further clarification and/or support. However, due to the rapidly evolving pandemic, these emails were unable to be sent the following day, as time-sensitive updates required immediate release. A Canadian participant articulated:

*I had a lot of questions [when I first read those emails]. I talked with my housemate and they did not know the answers either. We had to wait until the next day because all the staff were off. That was why every night [when the emails were sent], there was always some chaos happening on campus*.

One U.S. participant recalled, “*The university was sending emails*, *like three or four updates daily*, *like*, *what they were planning to do*. *[But] they weren’t sure about what to do*.” These machine-gun emails negatively affected the students.

One Canadian participant explained that “*Receiving so many emails together was very unusual*. *It made me so scared because I felt there might be some serious things that had happened*. *I felt like so many emails meant [the] university administration was in trouble*.*”*

Most participants’ experiences confirmed risk communication should avoid high frequency or repetitiveness. Student comments regarding high email frequency depicted that despite the emergency situation, emails should be administered normally, as high-frequency updates seemed to trigger campus-wide panic and even cause chaos.

### Element 2: Student interpretation

As previously mentioned, official emails should provide reliable information and resources to facilitate the student eviction process. The weaknesses identified in Element 1 created tremendous challenges for students in their efforts to correctly interpret the emails, even triggering some adverse coping-motivated behaviors.

#### Interpreting high email frequency

The university email administrative process aimed to soften the language. Almost all participants indicated eviction emails were written in very vague language. As an illustration, one Canadian participant described their interpretation of a gentle-toned, vague pre-eviction email: “*In-person classes were stopping*, *if you can leave*, *you can*, *but if you can’t*, *they’re not going to force you off-campus*. *It sounds like it is my personal choice*.” The ensuing emails, however, emphasized how swiftly the situation was evolving. One U.S. participant sought clarification from the student housing office; however, the participant was informed the office had received the same emails and could not provide further information. After several rounds of updates, another U.S. participant confirmed that communication would “*go back and forth for several rounds*, *it was pretty much just saying it’s best to leave*!”

One Canadian participant commented the emails only informed students of the university’s eviction decision but did not address concrete practical needs.

*[The eviction] emails like FYI [for your information], like here was the decision to move out. The university said in the emails that they would help us, like mental health support. However, no solid examples of what they could do, like storage space and mental health service were not even available*.

Most participants disclosed their universities did not provide students with sufficient essential support in conjunction with administrative eviction-related emails. One Canadian participant suggested that “*an online Q&A session would have been beneficial; it would be completely doable*.” When planning the eviction, another Canadian participant surprisingly discovered there was no official move-out policy available to quickly and safely guide their off-campus move, resulting in “*the dormitory look[ing] like a hurricane or an earthquake just hit it*.” This participant perceived that: “*If the university could have asked our needs in advance*, *or had some policy everyone could follow*, *[the results] would have been totally different*.” These examples further confirm the extreme events augmented and exposed related administrative process weaknesses.

#### Emotional reaction

This study reveals students’ emotional reactions towards eviction emails, and other administrative updates, sorted into the following three categories: ignoring, worrying, and complaining. Those who disregarded university emails and updates did not take immediate action, believing their peers were overreacting. These students did not seriously interpret potential COVID-19 risks, tending to hold beliefs similar to those who insisted coronavirus is “just like the flu” [64]. Those who worried, and those who carefully considered the eviction and the general COVID-19 situation, started to prepare for their eviction right away.

One Canadian participant had been anxiously monitoring COVID-19 related information since the outbreak began in China (December 2019). She described her classmate’s reaction: “*My labmate just cried when she read the email on her cell phone*. *I comforted her that we already knew it would happen*. *We were searching for new places immediately*.” This participant commented on some ignorers’ responses towards their concerted action: “*When some of our housemates saw us moving out*, *they just shook their heads and thought we were overreacting and [our move-out] was unnecessary*.”

According to Skubisz [65], risk increases with deeper emotional engagement (e.g., fear and anger). Participants observed extreme emotional peer reactions towards administrative emails, presented as excessive complaining and extreme anger; some of these students committed adverse coping-motivated behaviors across campus, such as throwing their personal belongings and roaring. These behaviors were repeatedly addressed in the daily updates circulated among all students. One Canadian participant expressed concern:

*Those emails just kept pointing out the students’ bad behavior almost whenever they happened and kept repeating it in almost all the emails. I did not think it was a good way to solve the problems. Students could copy the bad behaviors from others*.

A U.S. participant shared his thoughts regarding the repeated administrative statements concerning unfavorable student behaviors. “*They basically said that they couldn’t control what the students did; what they could control was what the university did—like the rules and regulations—because they didn’t want to be held liable as well*.”

As suggested above, some simple and feasible interventions (e.g., online Q&A sessions and proactively compiling student eviction-related practical needs) would have facilitated a smoother eviction process and might have reduced, or even avoided, unexpected negative coping behaviors. Administrative emails repeatedly drawing attention to negative behaviors might not effectively raise student awareness and generate movement toward order, as intended. Contrarily, they might augment behaviors, eventually jeopardizing on-campus community safety, solidarity, and cohesion. Universities should perceive destructive behaviors as red flags, warning administration to examine their emergency response plans, taking note of potential areas of improvement, especially pertaining to urgent services to fulfill frontline needs.

### Element 3: Interdepartmental collaboration

Inefficient COVID-19 responses among higher educational institutions worldwide have revealed a considerable number of institutions did not have sufficient basic emergency response plans in place [53]. This administrative limitation has directly elicited the ineffectiveness of interdepartmental risk communication, and the facilitation of other supplementary cooperation to advance on-campus eviction logistics, thus triggering extra burdens for students, as shown in the following three aspects.

#### Off-campus housing information

In response to the eviction order, moving to reliable off-campus housing became the most feasible solution, especially for international students who might be unable to immediately return to their home countries due to COVID-19-related international travel restrictions [57, 58]. Furthermore, many students in the first two years of their educational programs at Canadian institutions prefer on-campus housing [66]. In addition to safety and convenience considerations, these students may have selected on-campus housing as a protective steppingstone to develop various essential capacities required for independence. As some of these students may be living away from their families for the first time, they may not have sufficient local expertise to acquire off-campus accommodation and/or might not have yet developed the full capacity to verify related information.

One Canadian participant expressed: “*I directly moved into my student dorm from home and never looked for other off-campus housing*. *When I asked the student housing office [for off-campus housing information]*, *they suggested the online searching… Viewing became very difficult because I was afraid of getting infected while taking the bus*.”

Considering the prevalent on-campus housing shortage in North America, in conjunction with rapid student population growth, student enrollment departments typically collaborate with off-campus communities to provide reliable housing information to incoming students [67]. Overwhelmingly ineffective off-campus accommodation information service provision during the pandemic eviction process revealed unproductive interdepartmental cooperation, most notably between student enrolment and student housing departments.

One Canadian participant further elaborated: “*When I moved back home*, *about one week after [the deadline of] eviction*, *I received an email [from my university] about where to find places*. *Why didn’t they provide this information*, *early*, *like along with the eviction notice*?”

These examples demonstrate the importance of providing essential support, such as off-campus housing information, to coincide with emergency instructions, in this case, the campus-wide eviction order, to effectively address student needs in crisis situations. In addition to housing information, almost all participants insisted psychological support should have been offered in advance. A stable mental state would have established an emotional foundation for students to more accurately interpret administrative emails and better understand eviction plans.

#### Mental health support

Students typically experience increased academic pressure as a semester concludes. Additional eviction and pandemic-related emotional burdens aggravated their already stressed mental states, increasing mental health service demands. Two Canadian participants expressed concerns regarding on-campus mental health services.

*In the [eviction] email, a mental support phone number was included. I called this number [before the pandemic]. There was either a long time waiting, or they asked me to call back for daytime staff. I didn’t think it even worked during the eviction*.

*The night [when the eviction email circulated], throwing empty glass bottles, destroying dormitory facilities, and howling was going on. Just within hours, the campus security phone was ringing off the hook. But the security’s priority was not mental support. If there were some social workers, I think it would have been completely different*.

Emergency mental health care is one of the essential services typically offered during an emergency response [25]. During this emergency eviction process, the administration should have immediately secured and provided access to essential emergency services for the affected population. The unavailable urgent mental health services, and student adverse behaviors during the eviction process, exposed a lack of proper campus-wide interdepartmental cooperation.

#### Assistance program transparency

In March 2020, countries began releasing emergency financial assistance for their citizens [68]. Some community-based agencies also offered cash or in-kind support [69]. Accordingly, universities also designated emergency assistance programs [70]. To be effective and accessible, emergency assistance-related information must be disseminated widely; however, two Canadian participants revealed this relevant information was not circulated through their universities’ official channels.

*[Regarding university help], I was not aware of, like from friends or yet in emails… When I moved out, I did receive some free groceries from my community center*.

*I heard from the news that my university had some emergency funds for students. But I don’t think my university announced that*.

Although some resources were available, students confronted various barriers. A Canadian participant described his experience pursuing university assistance programs:

*I asked the student housing office. No luck at all. Then I went to the International Student Center [because I am an international student] and I was told that I was eligible for the emergency funds. This information was hidden. If I did not ask, I would never know. I shared it [with other international students]*.

Another Canadian participant, who “was very lucky” to receive some help from their university, clarified:

*I got an extension [for moving out] because I was an international student. My roommate [a domestic student] did not get that, and she had to fly from the east back home in the west. I know the International Student Center extended their office hours but did not serve the domestic students like my roommate*.

One U.S. participant doubted the university’s role during the eviction process:

*We pushed very hard. We asked [the university] for storage places and finally we got that. Then my university emailed us that the storage was available. I was so frustrated because even though some very basic things as storage places, if we did not push, we would have never gotten*.

Another Canadian participant provided suggestions for the university to help students to apply for government assistance:

*At first glance [of the email], I thought I was eligible. But all these programs had a lot of criteria. No contact information in the email for questions. I hope the university could provide some support, like the student center always makes email announcements that they could help, like applying for a student loan or visa application for international students*.

These examples indicate fundamental considerations regarding eviction logistics were initially neglected. As participants stated, they needed to “remind” the university of their essential needs. This initial inaction reveals administrative limitations regarding emergency response protocols, especially pertaining to fulfilling student-driven emergency needs.

### Element 4: Frontline voices

Merging Elements 1, 2, and 3 generates a new question for on-campus risk communication: how should administrators customize emergency messaging for their target audiences? In this project, target audiences pertain primarily to students who lived on campus and frontline staff who support on-campus residency operations. Engaging these two target groups might provide an effective approach to improve emergency mass email communication.

#### Focusing on urgent student needs

Inclusion is one of the universities’ primary communication principles [70]. However, in fulfilling this principle, at times, certain emails may not sufficiently address target group needs. On-campus resident students were one of the primary targets for eviction and related communications. One U.S. participant expressed: “*Most emails were sent out to everyone on campus… I believe the eviction order should have been composed for resident students only*. *This would have provided a better focus on our needs*.”

As previous examples demonstrate, essential logistical support and related services were not included with the first eviction email. Students questioned whether their universities developed eviction plans on behalf of students. One U.S. participant insisted:

*I felt the emails meant that ‘you [students] figure out your [students’] needs.’ Then they [university] tried to meet the needs. This might not be very helpful during the eviction. I was very surprised that my university, with over one-hundred-year history, had not had an emergency response plan*.

Most participants expressed compassion regarding their university’s challenges, particularly during this demanding period with enormous “first times” for all involved. However, when students pursued clarification and support from university authorities, they found ongoing challenges to be frustrating, increasing tensions within the existing student/university authority relationship. As the evictions exposed many areas for improvement within the universities’ emergency response planning, participants from both countries identified the take-away message from this experience as “*independence was key; you could not rely on your university*.” This message should provoke deep consideration among university authorities, as trust is central to risk communication and university administration, and this trust was fractured during the eviction process.

#### Engaging frontline feedback

Students highlighted some faculty member suggestions, which showed significant consideration and compassion for student practical needs.

One Canadian participant (graduate student) mentioned: “*My supervisor gave me several days off so that I could look for places*. *[The professor] told me that my department had emergency funds I could apply for and told me about some unsafe areas in town that I should avoid [when looking for housing]*.*”*

A U.S. participant in a design program recalled: *“Before the spring break*, *our professors told us to take everything with us because they didn’t really know what was going to happen*. *We need our supplies to continue with our degree*.*”*

Some university shutdowns took place over the spring reading break when some students were in the midst of traveling. These students needed to return to their campus(es) to collect their belongings. As public health protocols were in place, these students confronted additional challenges. A Canadian participant described car rental challenges relating to driving a great distance from home back to university to retrieve belongings:

*I needed to make an appointment, which was twenty minutes. Only [I] myself was allowed [in the building]. I lived on the fifth floor. I just put everything into storage boxes and dragged them downstairs. I needed to be very quick. Otherwise, I could not return the car on time, and the car rental company was on reduced service hours*.

A U.S. participant recounted:

*It was very stressful because I would have to fly all the way there, knowing I would be alone because it would be even more expensive to get one of my parents to go with me. I was just very stressed and overwhelmed because the university was kind of not giving us many options*.

Top-down communication strategies might not effectively embrace frontline voices. Of course, the time-sensitive nature of this emergency response might hinder the student feedback collection process; however, engaging student representatives in eviction planning and other emergency risk communication emails would be practicable. Furthermore, faculty members and frontline staff, who directly engage with student academic and social activities, would be valuable resources in this process. Engaging various front-line perspectives would strengthen risk communication, improving the eviction process overall.

## Discussion

**Fig 2:**
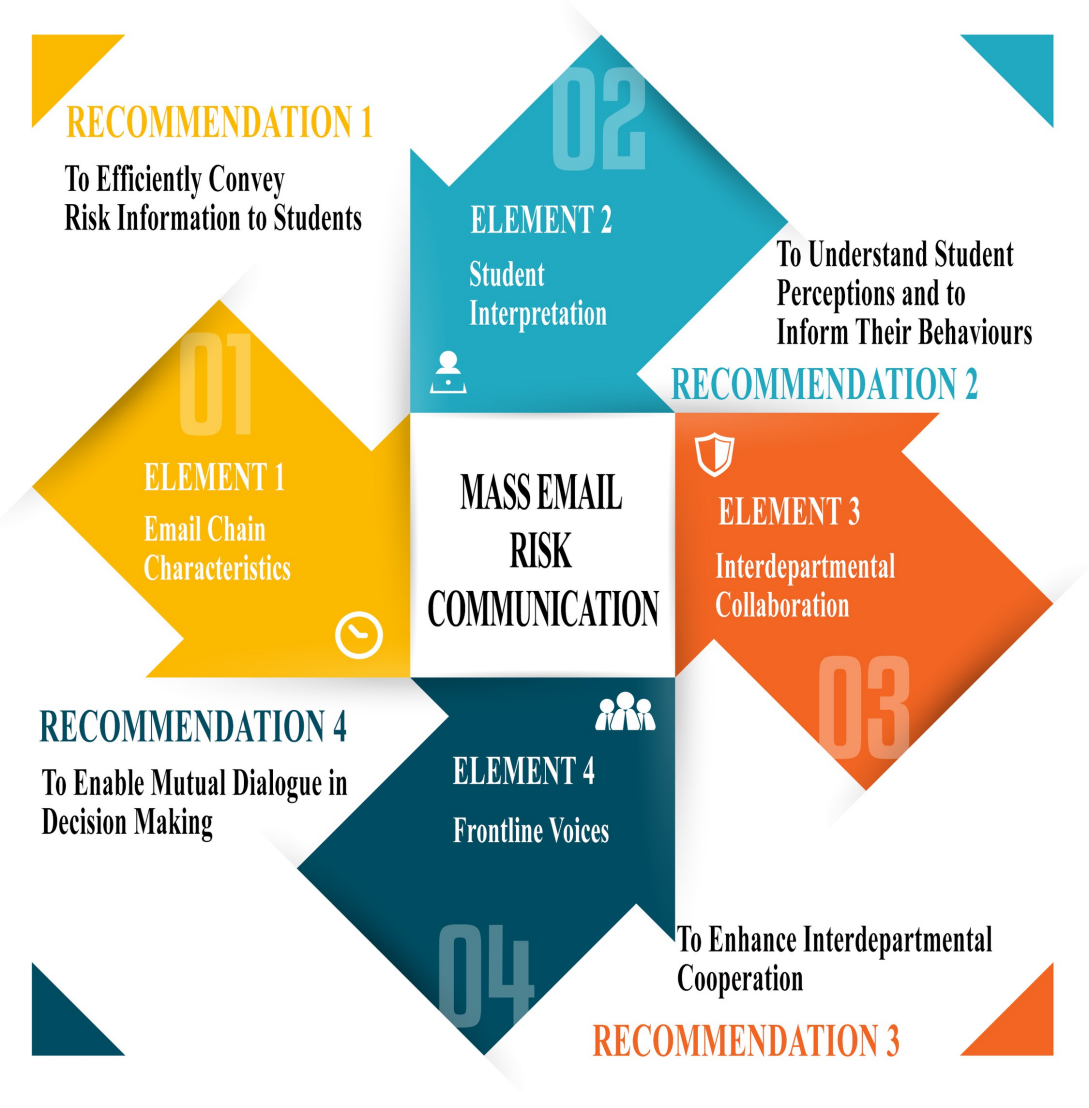
Mass email risk communication: Elements and recommendations. The arrows present the four themes from the finding section. In responding to each theme, a recommendation was developed.

In a post-secondary educational context, email is a widely used approach to communicate with a large group of employees and enrolled students [12]. Serving as the primary risk communication approach, these mass emails must inform at-risk students, faculty, and staff to protect themselves, prepare, and take appropriate actions. The four themes presented above, however, demonstrate that incomplete and/or unsuitable email composition, and subsequent inefficient follow-up services places additional burdens upon the majority of recipients and hampers important messaging intended to provide situational guidance and support on-campus dweller health and well-being. Synthesizing these themes builds evidence-based recommendation guidelines for on-campus community mass email risk communication (see Fig 2).

### Recommendation 1: To efficiently convey risk information to students

During the eviction process, the participant-identified long, gently worded emails with inclusive language may have served to relieve evictee anxiety; however, as email length increases, opportunities for recipient uncertainty and confusion also rise. As email interpretation varies between recipients, a brief and to-the-point message might avoid negative consequences by answering the following questions: What are the current risks? How did these risks develop? What are the university’s expectations? How could the university work with students to achieve these expectations?

The swiftly evolving COVID-19 pandemic illustrates that as new information comes to light, emergency instructions may change quickly, and at times, immediately. In this novel context, it is understandable administrative mass-emails contained uncertain and inconsistent messaging. When uncontrollable factors arise, brief explanations could serve well to alleviate student concerns. As students sought to balance their educational process with potential pandemic risks, brief descriptions would more effectively support student decision-making. Furthermore, transparency is recommended in terms of salient information directly affecting the eviction process (e.g., what are the move-out extension application criteria?).

Daily updates could promptly inform students of important information; however, high frequency and redundancy contribute to possible outcomes contrary to risk communication goals: increased confusion and anxiety, and students ignoring administrative emails. As mentioned above, individual email interpretation varies [51]; therefore, questions and requests for further clarification are unavoidable. To mitigate this concern, intentionally planned midday administrative email updates would enable students to seek clarification with staff during their regular working hours.

### Recommendation 2: To understand student perceptions and to inform their behaviors

In a complex COVID-19 environmental context (e.g., anti-mask rallies as well as political drama and propaganda), participants experienced tremendous challenges to salient information identification. In these conditions, critical information could be easily overlooked, downplayed, or even misinterpreted, leading students to overestimate potential eviction-related negative impacts, possibly triggering overwhelm, depression, and anxiety. Some students (the ignorers) underestimated COVID-19 and the eviction, whereas some (the worriers) were extremely concerned and took immediate action upon eviction email receipt. Effective email communication, at this point in an emergency, should raise ignorer awareness, reassure worriers, and avoid galvanizing complainers.

Complainer coping-motivated adverse behaviors might not be entirely preventable during the eviction process. Risk communication, however, can responsibly issue warnings to reduce adverse behavior frequency, instead of encouraging actions that generate mutual benefit. Emails should be carefully crafted to both sharply denounce these destructive behaviors and raise student situational awareness, with the intention to avoid repeating or augmenting the unfavorable behaviors. The administration must be mindful that repetitious emails may further incite the rebellious mood, possibly escalating the adverse behaviors, rather than achieve sender intent: to successfully educate target audiences—both the students who committed destructive behaviors and also other on-campus community dwellers.

### Recommendation 3: To enhance interdepartmental cooperation

Correctly executed mass email risk communication not only fulfills the university’s role as a reliable information hub, but also elucidates the need for universities to support on-campus dwellers in emergencies. Hence, along with eviction notices, related support and services are required. Students identified timely psychological support and eviction logistics as the most urgently required supportive considerations (e.g., off-campus housing information, storage facilities, and simple moving equipment). In a typical academic year, such supportive services could be smoothly and successfully administered, providing students with timely assistance through interdepartmental cooperation. However, COVID-19 stressed existing systems, presenting greater organizational requirements and unexpected additional challenges, testing internal collaborations.

The pandemic exposed interdepartmental cooperation weaknesses within university systems, generating an opportunity for improvement. The projected best-case scenario guarantees essential supportive services are available, accessible, and dependable when extreme events arise. Moreover, supportive services and related information must be widely circulated amongst the student population. Providing supportive services also introduces a powerful strategy to re-establish trust between students and university authorities, repairing damaged relationships potentially triggered through administrative missteps during the initial emergency response.

### Recommendation 4: To enable mutual dialogue in decision making

As resident students are among the primary on-campus eviction email recipients, risk communication strategies should include customized student-specific messages. This would signify a willingness to support students with specific strategies, rather than obligating students to depend upon their personal connections and resources to coordinate their eviction processes, or rely on students to ad hoc assist university authorities in identifying their needs. Roleplaying as students would allow university authorities to understand student eviction-driven needs and provide solutions accordingly.

As mentioned above, mutual dialogue is critical for risk communication during an emergency response [47]. Emergencies, however, present extraordinary circumstances that might limit regular dialogues with frontline stakeholders due to time pressures and other novel challenges. As students proposed, hosting virtual Q&A sessions, consulting frontline faculty and staff, and engaging student representatives in decision-making could, in the future, be implemented. Furthermore, intentionally developing and practicing a means for university administrators to strengthen student-needs understanding during non-emergency times, would enable swift knowledge transfer into emergency scenarios as they arise.

## Limitations and future research directions

The findings are impressive; however, limitations are outstanding. For instance, the author’s professional background has influenced the data interpretation. As a mitigation strategy, the author conducted two rounds of data analysis, but recused himself during each round to ensure the data analysis was grounded in participant experiences. Additionally, the following three limitations were identified, which also suggest future research directions.

To begin, during the interview process, participants potentially intuitively compared university email risk communication during the on-campus eviction period with standard administrative emails received in the period before COVID-19. This type of informal comparison infers some basic information regarding university email communication pre-COVID-19; however, a systematic review of mass eviction-related emails is needed to support a comprehensive understanding of the entire email risk communication landscape at participant universities. This understanding would advance data analysis by identifying background information and any unique requirements each university authority addressed.

Furthermore, as addressed in the literature review sections, although not all university/college authorities have comprehensive emergency response strategies (including a risk communication component), each university/college should have email communication regulations and policies in place. Reviewing related regulations and policies would contribute to understanding institutional email communication strategies to enhance data analysis and enable the development of university-specific recommendations to improve mass email risk communication within their emergency response efforts. Future research might use a case study approach to review one university/college’s email communication regulations and responsively explore student reactions toward mass email risk communication. This approach will effectively identify areas for improvement and develop related solutions.

Moreover, demographic factors such as gender, ethnicity, and immigration status influence emergency behaviors [10]. Although both Canadian and U.S. research participants share some similarities and present tremendous differences [71], all of which provide rich background information to support data analysis, country-based differences did not significantly emerge among mass email communication themes. In order to protect participant privacy and confidentiality, only a few demographic variables were collected. Due to the limited sample size, the study cannot comprehensively contextualize participant experiences with their demographic factors. Future research may apply a targeted approach to recruiting participants from certain demographic groups to examine related influences. A quantitative approach could also contribute to measuring the correlations between different demographic factors and eviction experiences.

## Conclusion

The delivery of correct information to the right person at the right time is vital in risk communication [72]. Based on the COVID-19-triggered campus-wide evictions in Canada and the U.S., this study qualitatively identified the following areas for improvement within mass email risk communication. Outgoing administrative mass emails should convey brief, consistent, and transparent risk information to facilitate student eviction decision-making by enhancing their perceptions and informing their coping behaviors. University authorities should bear the fundamental responsibility to guarantee essential supportive service availability, accessibility, and dependability. Information concerning these services must be disseminated widely to benefit the most significant number of students. Furthermore, mutual dialogue between the university and students should be conducted in both emergency and non-emergency scenarios to properly assess student needs and build appropriate comprehensive strategies to improve eviction experience outcomes. These evidence-based recommendations could directly assist post-secondary educational organizations in strengthening their risk communication strategies and advancing their emergency response plans.

The global COVID-19 pandemic has reiterated that every community needs to prepare for potential emergencies. Risk communication is the first and foremost emergency response measure. Within the on-campus community, in addition to delivering critical information, risk communication also assumes the responsibility of raising student and stakeholder risk awareness, reducing anxiety, encouraging protective behaviors with mutual benefits, and managing unfavorable outcomes. Furthermore, at its best and most productive, mass email risk communication focuses on and coordinates on-campus resources to promote healthy dialogue, engage students in decision-making, and strengthen connections among different departments—both within the same university system and between university authorities and their students. Applied in educational settings in particular, these strategies would serve to improve mass email risk communication, and if applied in a more general context, these strategies may also advance mass email risk communication and emergency response planning across organizational domains.

### Institutional review board statement

The study was conducted according to the guidelines of the Declaration of Helsinki, and approved by (1) the Social Sciences and Humanities Research Ethics Board at Dalhousie University, Canada (Certificate Number: 2020–5371) and the informed verbal consent was obtained from each Canadian participant; and (2) the Institutional Review Board at the University of Puerto Rico Mayaguez, the U.S. (Protocol Number: 2020040014), which waived the requirement to obtain informed consent for all the U.S. participants.
